# The Impact of Nasogastric Tubes (NGTs) on Swallow Function in Adults with Post-stroke Dysphagia: A Systematic Review

**DOI:** 10.1007/s00455-026-10926-7

**Published:** 2026-03-24

**Authors:** Layal Hanna, Zaneta Mok, Lital Weizman, Angela Burge, Cassie McDonald

**Affiliations:** 1 Department of Speech Pathology, Alfred Health, Melbourne, Australia; 2 School of Allied Health, Human Services and Sport, La Trobe University, Melbourne, Australia; 3 Department of Physiotherapy, Alfred Health, Melbourne, Australia; 4 School of Translational Medicine, Monash University, Melbourne, Australia; 5 Department of Physiotherapy, The University of Melbourne, Melbourne, Australia; 6 The University of Melbourne, Level 7, Alan Gilbert Building, Barry St, Carlton 3052 Melbourne, Victoria Australia

**Keywords:** Nasogastric tube, Stroke, Dysphagia, Swallowing, Enteral nutrition, Aspiration, Rehabilitation

## Abstract

Nasogastric Tubes (NGTs) are a first-line treatment for delivering short-term nutrition to stroke survivors with dysphagia. However, the impact of NGTs on swallowing function itself is not yet well understood, despite the clinical importance of understanding this relationship to guide NGT treatment decisions. This review examined current evidence on the effects of NGTs on swallowing function in this population, focusing on five outcomes of interest: overall swallowing function, penetration/aspiration, oral and pharyngeal transit times and pharyngeal residue. A systematic search of terms related to stroke, dysphagia and NGTs across four major databases identified 1099 records. Eight full-text articles met inclusion criteria, of which seven were a pre-post design and one was a randomised control trial. Sample sizes of included studies ranged from 21 to 147 participants and median ages were between 66.0 ± 8.5 to 73.2 ± 12.4 years. Other participant characteristics, such as time with NGT in situ, time since stroke and NGT type, were inconsistently reported. Six studies used an instrumental swallowing evaluation to evaluate change between conditions, however, significant methodological heterogeneity between studies was evident in the inclusion criteria and assessment methods reported. Data synthesis suggested the following trends when an NGT is present: greater overall dysphagia severity, longer pharyngeal transit times, increased severity of penetration/aspiration, higher rates of pneumonia and increased volume of pharyngeal residue. However, most studies had a moderate-high risk of bias (*n* = 7), limiting the strength of conclusions. More methodologically robust research is needed to clarify the impact of NGTs on swallowing physiology and inform clinical management of dysphagia in stroke survivors requiring an NGT.

**Supplementary Information:**

The online version contains supplementary material available at 10.1007/s00455-026-10926-7.

## Introduction

 Oropharyngeal dysphagia, or impaired swallowing, is a common consequence of stroke with potentially significant consequences. Reports of the incidence of post-stroke oropharyngeal dysphagia have ranged from 8 to 80% [1, 2] due to variation in diagnostic tools, stroke characteristics and included participants; however, a recent systematic review estimated a pooled incidence of 42% [2]. In severe cases of dysphagia, nasogastric tubes (NGTs) are used to deliver adequate short-term nutrition, with a recent large retrospective cohort study finding that up to 23% of stroke survivors with dysphagia required NGT or another form of enteral feeding throughout their hospital admission [3]. Prolonged dysphagia significantly increases mortality risk after stroke [6]. One study found patients with dysphagia are 8.5 times more likely to die post stroke than those without [4], with the risk further increased for stroke survivors requiring NGTs [4].This higher mortality is likely due to a higher incidence of acquired complications such as aspiration pneumonia [2, 4] and malnutrition [5], both of which contribute to longer hospital stays [6] and higher healthcare costs [7, 8] compared to stroke survivors without dysphagia.

These complications are usually caused by impaired swallowing safety (i.e., airway protection) and/or efficiency (i.e., movement of the bolus towards the oesophagus with minimal effort or residue) [11]. For example, impaired airway protection can result in penetration and/or aspiration, increasing the risk of total airway obstruction or aspiration pneumonia. Furthermore, inefficient swallowing (due to poor bolus containment and control, prolonged mastication, slowed transit through the oropharynx and significant post-swallow residue in the oral/pharyngeal cavities) can result in inadequate nutrition and hydration. For patients with severely impaired swallowing safety or efficiency, NGTs are an essential tool used to bypass the need for swallowing and ensure adequate nutritional intake [9, 10]. As swallowing function improves, reliance on the NGT typically decreases until the tube is no longer needed [11].

However, NGTs rest against the posterior pharyngeal wall, a structure critical for both airway protection and bolus propulsion towards the oesophagus. The tube’s position may interfere with these mechanisms by impacting the strength or length of muscle contractions in the pharyngeal wall, potentially resulting in a higher likelihood of pharyngeal residue or penetration/aspiration [9]. This is consistent with evidence indicating proportionally higher rates of aspiration pneumonia in dysphagic patients with NGTs compared to other forms of enteral nutrition [10], suggesting that airway protection is more compromised in patients with a NGT. However, these impacts on swallowing physiology are not yet well understood. This information is critical to ensure that treatments for both nutrition and swallowing are carefully balanced. In order for clinicians to prescribe an appropriate dysphagia treatment plan, they must first understand the physiological swallowing impairments [11]. Therefore, it is essential to determine whether observed swallowing impairments stem from stroke-related neuromuscular dysfunction or are due to the mechanical presence to the NGT and likely to resolve spontaneously upon its removal. Conversely, if impairments persist beyond NGT removal, it is critical to determine the need for additional rehabilitation to mitigate lingering effects of the NGT.

To our knowledge, no systematic reviews have been conducted on this topic and fewer than 15 primary studies have directly compared swallowing physiology with and without an NGT in place. These studies have largely involved either healthy participants or individuals with dysphagia of mixed aetiologies and have varied significantly in study design and overall findings. Huggins et al. [12] found slower swallowing transit times with wide-bore NGTs in healthy younger adults (*n* = 10, age range 20–27 years) compared to no NGTs. Pryor et al. [13] conducted a similar comparison and reported delayed pharyngeal transit and increased penetration/aspiration in healthy older adults with both fine- and wide-bore NGTs (*n* = 9, median age 65 years) compared to no NGTs. These findings suggest there may be age-related differences in the ability of healthy adults to swallow with an NGT in situ, and that the width and consistency being trialled may also affect swallowing. However, due to the small samples sizes of these two studies, the results must be interpreted with caution. Regardless, the implications are important: if the presence of an NGT impairs swallow in healthy individuals, it may be posited that this is compounded for persons with dysphagia.

Leder et al. [14] and Fattal et al. [15] both examined patients with dysphagia, including those post-stroke (*n* = 630 and *n* = 62 respectively) and found no significant differences in aspiration rates based on sex, age, or dysphagia cause. Whilst existing literature suggests that an NGT may not be associated with an increased incidence of aspiration, its impact on other aspects of swallowing (such as timing and efficiency) remains unclear.

To our knowledge, this is the first systematic review to examine the current evidence regarding the impact of NGTs on swallowing function and critical aspects of swallow physiology (such as airway protection, efficient bolus transit and effective bolus clearance) in stroke survivors. The authors hypothesise that due to the position of the NGT within the pharynx, swallowing function could be negatively impacted in patients with post-stroke dysphagia.

## Method

This review was prospectively registered on the PROSPERO International Registry of Systematic Reviews (registration number: CRD42023377722). The methodology and reporting of results followed the PRISMA guidelines [16].

### Search Strategy

A comprehensive literature search strategy was developed in consultation with a health sciences librarian (LR) and carried out in January 2023. Electronic bibliographic database searches were conducted in EMBASE, EMCARE, Medline and CINAHL (Appendix 1). The reference lists of the included articles were also screened by hand for additional references.

### Study Selection

Studies were included according to the following criteria:

- Published in English.
- Type of studies: presence of a control or comparison (between or within-group) to evaluate the effect of NGT (for example, NGT compared to no-NGT at two time points or across two groups).
- Participants: aged ≥ 18 years; ≥50% of included participants had post-stroke dysphagia.

The primary outcome of interest for this study was overall swallowing function, operationally defined as a measurable change in one or more swallowing-related parameters, as quantified by the assessment instrument or tool employed within each individual study. Secondary outcomes of interest were:

- Swallowing safety:
  - Laryngeal penetration: Material that enters the larynx before, during or after swallowing but remains above the vocal folds. Can be present in healthy older adults with thin fluids or mixed consistencies, however otherwise considered abnormal [9, 17].
  - Aspiration: Material that enters the larynx before, during or after the swallow and passes through the vocal folds into the trachea; considered abnormal [9, 17].
- Swallowing efficiency:
  - Swallowing transit times: The number of seconds taken for the bolus to be transferred and swallowed. This can be further broken down into oral transit time (OTT; time between first bolus movement in the mouth until the bolus crosses the lower rim of the mandible) and pharyngeal transit time (PTT; time between the triggering of the pharyngeal swallow to when the bolus tail passes through the upper oesophageal sphincter) [17–19].
  - Pharyngeal residue: Material that remains in the pharynx after the completion of the swallowing sequence, typically observed in the vallecular cavity and/or the pyriform sinuses. In healthy adults, residue is rare or minimal [17, 20].

Only primary studies employing quantitative methods with extractable quantitative data were included as the authors aimed to complete a meta-analysis if appropriate. Therefore, conference papers, tutorials, reviews, and qualitative studies were excluded. All studies identified by the search were uploaded to Covidence systematic review software and duplicates removed [21]. Two independent reviewers screened titles and abstracts for eligibility then reviewed full texts of the qualifying studies according to inclusion criteria. Disagreement during screening and full text study selection process was resolved by discussion and consensus. A third reviewer was available for consultation if consensus could not be reached, however, this was not required.

### Quality Assessment

Two reviewers completed quality appraisal of the included studies using the relevant Johanna Briggs Institute (JBI) critical appraisal checklist according to study design [22]. A third reviewer was consulted when consensus could not be reached. The JBI checklist for Randomised Control Trials (RCTs) included an assessment of the risk of bias due to group allocation (randomisation and concealment); blinding (participants, therapists, and outcome assessors); and selective outcome reporting. Similarly, the JBI checklist for case-series included analysis of outcome measurement and comparison. Questions were categorically scored as “yes” or “no,” and following consensus, these categorical ratings were converted to numerical scores (1 = yes, 0 = no). The maximum available score was ten for case series studies and 13 for RCTs. As there is no published standardised interpretation for the JBI checklist, cut-off values for bias levels were determined by the research team, as recommended by the JBI manual [23]. Thresholds were based on a similar approach used in previous research [24]: low risk of bias if ≥ 70% of answers scored yes, moderate risk if 50–69%, and high risk if < 50%. The percentage of agreement between reviewers was recorded. Rather than relying solely on an overall score, results were presented in a tabulated format, as recommended by the JBI manual. This allowed for a more detailed interpretation of the findings and provided context for the use of predefined thresholds and a binary scoring approach.

### Data Extraction & Analysis

A standardised data extraction form was piloted with two articles and no changes were made. Data were extracted from included studies by two reviewers working independently . The two datasets were then compared for discrepancies which were resolved by consensus discussion. Data were extracted under the following headings: study setting; study population and participant demographics and baseline characteristics (e.g., time since stroke, length of time with NGT in situ etc.); details of the NGT intervention and control conditions; study design; study methodology (e.g., type of swallowing assessment used, time between assessments in single group studies); outcomes, and suggested mechanisms of NGT intervention. As the included studies were clinically heterogeneous [25], narrative synthesis was used conducted according to the Synthesis Without Meta-analysis (SWiM) guideline [26]. Data from RCTs and non-randomised studies were not combined for analysis. Study results were grouped according to the key outcomes defined above to allow for systematic synthesis and comparison of the results. Where data was available, raw mean differences and associated confidence intervals were calculated.

## Results

### Summary of Included Studies

**Fig. 1:**
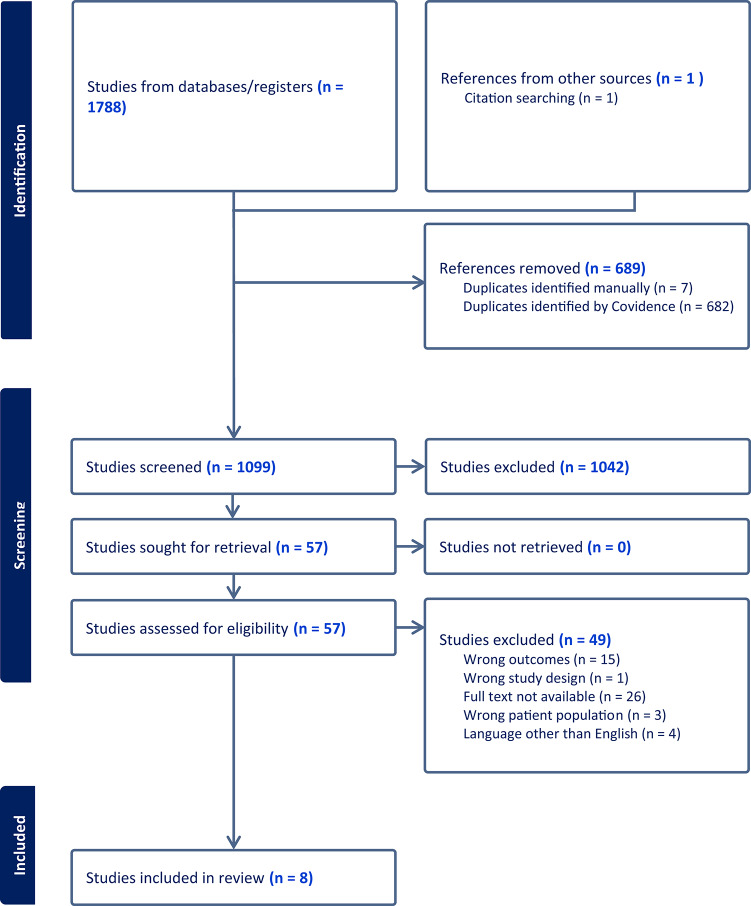
PRISMA diagram summarising study identification, screening, and inclusion

Figure 1 shows the PRISMA diagram summarising the search strategy and results for this study. A total of eight studies met the inclusion criteria.

### Study Characteristics

All studies were single-centre designs conducted across Germany [27], Taiwan [28], China [29, 30] and South Korea [31–34]. Seven studies had a pre-post design whereby the same participants were assessed twice, once with the NGT inserted and once with it removed. One randomised control trial (RCT) that compared outcomes between NGT feeding and intermittent oro-oesophageal tube feeding (a feeding tube that is inserted into the oesophagus via the mouth during feeding times and removed between feeds) was included [29].

Participant selection criteria and recruitment methods varied substantially across the eight studies. Only two studies [27, 33] provided data on how dysphagia was identified in the included participants, and only one of which utilised a validated screening tool [27]. The remaining studies lacked explicit diagnostic criteria for dysphagia, reporting only that patient recruitment was based on referrals to either Speech Pathology, rehabilitation, or dysphagia clinics [28–30] or identified through retrospective reviews of medical records conducted within a specified timeframe [31, 32]. Six studies excluded patients who were medically unstable or had confounding medical conditions, such as heart failure, myasthenia gravis, pneumonia or other systemic infection at the time of the study [28–33]. Five studies excluded patients who were unable to follow directions due to decreased conscious state or low cognitive function [28–32]. Two studies excluded patients with a prior history of surgery and or structural abnormalities the oropharynx or oesophagus, such as head and neck cancer, thyroid surgeries, vocal cord palsies and strictures [28, 30]. Three studies excluded participants with aspiration, though the specific criteria differed significantly. This exclusion appears to have been made to minimise medical risk during study participation. Wang, Chen and Ni [30] excluded patients who demonstrated any aspiration on VFSS assessment, whereas Wang et al. [28] and Nam et al. [34] excluded patients who had ‘more than a minor amount of aspiration’ (defined as ‘sufficient to form a continuous coating on the tracheal wall’) and ‘severe aspiration’ (defined as a rating of greater than five on the modified Penetration-Aspiration Scale [35]) respectively. Jung et al. [31] and Kim et al. [32] did not report any additional selection criteria beyond presence of an NGT and the time within which participants were assessed.

### Participant Characteristics

Table 1 summarises the salient participant characteristics reported within the studies. Notably, sample size justification and power calculations were rarely reported (*n* = 1) [29]. Where data of interest was not reported, the three relevant authors were contacted for further information between July and September 2023, however none responded despite an initial email request, and one follow up email request over 12 months.

**Table 1:** Summary of reported participant characteristics

Author, year	*n*	Participants with stroke (%)	Age (years)	Sex (% female)	Time with NGT in situ	Time since stroke	NGT type (Fr)
*RCT*							
Juan et al., 2020 [29]	97	100	control 66.2 ± 8.5	44	28.9 ± 4.9 days	Not reported	Not reported
			intervention 66.0 ± 9.1	39			
*Case series*							
Wang et al., 2006 [28]	22	100	69.7 ± 17.8	46	> 2 weeks	20.3 days	16
Dziewas et al., 2008 [27]	25	100	73.1 ± 13.9	53^a^	Not reported	3.6 ± 2.1 days	14 or 16
Wang, Chen and Ni, 2019 [30]	30	100	71.4 ± 4.1	43	69.5 ± 7.7 days	Not reported	14
Kim et al., 2018 [32]	147	66	74.2 ± 9.2	38	Not reported	Not reported	16
Kwak et al., 2018 [33]	70	64	A (stroke patients with NGT): 65.2 ± 14.9	35	Not reported	1.9 ± 1.8 years	Not reported
			B (control group: stroke patients without NGT): 65.3 ± 13.5	52			
			C (healthy adults without stroke or dysphagia): 60.1 ± 13.4	44			
Nam et al., 2015 [34]	40	60	63.3 ± 17.0	48	106 days (SD not reported, range 6–882)	Not reported	18
Jung et al., 2021 [31]	21	57	73.2 ± 12.4	67	Range 1–23 weeks	2 weeks – 13 years	Not reported

### Methodological Quality of Included Studies

The quality of the evidence was mixed. The only RCT [29] included was assessed to be at a low risk of bias [29] (Table 2). Of the seven non-randomised studies, five were assessed to be at a moderate risk of bias [28, 30, 32–34] and the remaining two pre-post designs [27, 31] assessed as being at a high risk of bias (Table 3). Most studies included appropriate statistical analysis (*n* = 6) [29–34] and clearly reported outcomes and demographic information (*n* = 7) [27, 28, 30–34]. However, only two studies reported how participants with dysphagia were identified or how dysphagia was diagnosed [27, 33], and only one of which used a validated diagnostic tool [27].

**Table 2:** Results of risk of bias analysis: JBI checklist for RCTs

	True randomisation used?	Allocated to treatment groups concealed?	Treatment groups similar at baseline?	Participants blind to assignment?	Those delivering treatment blinded?	Treatment groups identical at baseline?	Outcome assessors blind to treatment assignment?	Outcomes measure in the same way between groups?	Outcomes measured in a reliable way?	Follow up complete?	Participants analysed in groups to which they were randomised?	Appropriate statistical analysis used?	Trial design appropriate?	Overall appraisal (score) (score, risk of bias)	Risk of bias
Juan et al., 2020 [29]	Yes	Yes	Yes	No	No	Yes	No	Yes	Yes	Yes	No	Yes	Yes	76%	Low

**Table 3:** Results of Risk of Bias analysis: JBI Checklist for Case Series and non-RCTs

Case series	Clear criteria for inclusion in the study?	Condition measured in a standard, reliable way for all participants?	Valid methods used for identification of the condition for all participants?	Consecutive inclusion of participants?	Complete inclusion of participants?	Participant demographics clearly reported?	Clear reporting of clinical information of participants?	Outcomes clearly reported?	Clear reporting of site/clinic demographic information?	Appropriate statistical analysis?	Overall appraisal (score)	Risk of bias
Jung et al., 2021 [31]	Unclear	Unclear	Unclear	Unclear	Unclear	Yes	No	Yes	No	Yes	30%	High
Dziewas et al., 2008 [27]	Yes	Yes	Yes	Unclear	Unclear	Yes	No	Unclear	Unclear	Unclear	40%	
Wang et al., 2006 [28]	Yes	Unclear	Unclear	Unclear	Unclear	Yes	Yes	Yes	Yes	No	50%	
Kim et al., 2018 [32]	No	Unclear	Unclear	Yes	Yes	Yes	No	Yes	Unclear	Yes	50%	Moderate
Nam et al., 2015 [34]	Yes	Unclear	Unclear	Unclear	Unclear	Yes	Yes	Yes	Yes	Yes	60%	
Kwak et al., 2018 [33]	Unclear	Yes	Unclear	Unclear	Unclear	Yes	Yes	Yes	Yes	Yes	60%	
Wang, Chen and Ni, 2019 [30]	Yes	Unclear	Unclear	Unclear	Unclear	Yes	Yes	Yes	Yes	Yes	60%	

#### Swallowing Assessment Methodology

##### Assessment Type

Studies varied as to how swallowing assessments were conducted (Electronic Supplementary Material 1). Six studies used an instrumental swallowing evaluation such as videofluroscopic swallow studies (VFSS) or fibreoptic evaluation of swallowing (FEES), which allowed for direct visualisation of the swallow and movement of the bolus through the pharyngeal +/- oral cavities [27, 28, 30–32, 34]. Other assessment methods included the Wa Tian Water Drinking Test [29] (participants drank 30mL warm water whilst assessors recorded behaviour such as swallowing time and coughing) and ultrasonography to indirectly assess swallowing by measuring changes in hyoid movement during the swallowing sequence [33].

##### Outcome Measures

Studies used a variety of outcome measures to measure swallowing changes, as detailed in Electronic Supplementary Material 1.

##### Bolus Type and Volume

There was considerable methodological heterogeneity in the bolus types and volumes used not only between studies but also within studies. Four studies tested one bolus type [29, 32–34] and the remaining four tested at least two [27, 28, 30, 31]. Bolus volume ranged between 1mL to 30mL. Bolus viscosity was consistently reported, however only one study only one study [30] referenced the relevant 2012 International Dysphagia Diet Standardisation Initiative (IDDSI) levels [36] making comparison across studies difficult. Five studies specified ‘thin fluids’ or ‘water’ and three studies [27, 28, 31] specified ‘puree’ or ‘paste’ presumably equating to IDDSI Levels 0 and 4 respectively. The other viscosities were unable to be approximated to relevant IDDSI levels as insufficient detail was provided. For example, one study [28] trialled ‘thick barium’ but it was unclear if this was IDDIS Level 1, 2 or 3, another [37] trialled soft solids but it was unclear if this was IDDSI Level 5 or 6. In some cases, not all participants within the same study received the same bolus types. For example, one study reported that all participants (*n* = 25) received ‘puree’, but only 21 received ‘liquids’ and 18 received ‘soft solids [27]. Similarly, Wang et al. [28] reported that participants in the ‘with NGT’ condition only were given a barium ‘paste’ in addition to the ‘thin’ and ‘thick’ barium provided in the ‘without NGT’ condition.

##### Timeframe between Pre and Post Evaluation

Of the seven studies with pre-post design, five studies reported on the time between measures [27, 28, 30, 31, 34] but was not reported in the two remaining studies [32, 33] (Electronic Supplementary Material 2).

#### Outcomes of Interest

Effect sizes were not reported in the included studies; confidence intervals and the statistical significance of reported findings are provided where available.

##### Overall Swallowing Function

Four out of the eight studies included a comparison of overall swallowing function between the two conditions (NGT and no-NGT) [29–31, 34]. Of these, two studies used the FDS [30, 34] and one used the VDS [31] (Table 4). All three studies reported a higher mean score for the ‘NGT’ condition for at least one bolus viscosity, indicative of a greater swallowing impairment. The remaining study reported a statistically significant difference across all five levels of the Wa Tian Drinking Water Test at the end of the intervention period between the intervention group who received intermittent oesophageal tube feeding and control group who received continuous NGT feeding (Z = 2.496, *p* = 0.013); improvement in swallowing function was found in 83.67% of participants with the intermittent oesophageal tube feeding (intervention) condition compared to 66.67% of participants allocated to the NGT feeding (control) group (x^2^ = 15.574, *p* < 0.001) [29].

##### Oral Transit time (OTT) and Pharyngeal Transit time (PTT)

Three studies measured OTT and PTT (Table 5) [28, 30, 34]. Overall, no differences were found in OTT between the two conditions in either of the three studies. PTT results were variable; one study found increased PTT [30] and the other did not [28].

**Table 4:** Summarised results of studies using Functional Dysphagia Scale or Videofluoroscopic Dysphagia Scale to assess change in overall swallowingfunctionSummarised results of oral transit time and pharyngeal transit time

Author, year	Bolus Type	NGT	No NGT	*P*-value	Mean difference (95% CI)
*Functional Dysphagia Scale*					
Wang, Chen and Ni, 2019 [30]	Thin barium	28.07 ± 5.01	12.20 ± 4.39	< 0.001*	15.87 (13.44; 18.30)
	Thick barium	30.23 ± 6.09	13.93 ± 6.03	< 0.001*	16.3 (13.17; 19.43)
Nam et al., 2015 [34]	35% diluted barium solution	33.96 ± 16.20	26.76 ± 15.00	< 0.001*	7.20 (0.25; 14.15)
*Videofluoroscopic Dysphagia Scale*					
Jung et al., 2021 [31]	Nectar	32.6 ± 16.1	30.9 ± 16.6	0.033*	1.70 (-8.50; 11.90)
	Puree	31.4 ± 15.5	30.4 ± 15.8	0.114	1.00 (-8.76; 10.76)

Data are mean±SD total score unless indicated. NGT, nasogastric tube

##### Penetration/aspiration

All studies included outcomes relating to penetration-aspiration [27–34], two of which excluded participants with ‘severe aspiration’ as discussed previously [30, 31]. Most studies (*n* = 6) directly measured the presence, absence or degree of penetration-aspiration using an instrumental swallowing assessment (FEES or VFSS) [27, 28, 30–32, 34]. Three of these provided data on means and mean differences on the PAS or mPAS (Table 6) [30, 31, 34]. The remaining three studies reported descriptive statistics which did not show change between the conditions [27, 28, 33] Two studies reported on indirect indicators of aspiration and aspiration risk. One RCT compared patients fed with an NGT with patients fed with an intermittent oro-oesophageal tube and found increased rates of pneumonia in the NGT group (x^2^ = 12.866, *p* < 0.001) [29]. A within-group comparison found that stroke patients with an existing NGT had reduced degree (*p* = 0.005) and reduced distance (*p* = 0.009) of hyoid bone movement in comparison to when the NGT was removed [33]. Similarly, differences in hyoid movement were found when stroke patients with an NGT were compared to two control groups: stroke patients without an NGT and healthy adults without stroke or dysphagia (*p* < 0.001). Furthermore, hyoid movement in stroke patients with an NGT was restored back to the level of stroke patients without an NGT once their NGT was removed (mean = 0.3, SD = 0.8).

**Table 5:** Summarised results of oral transit time and pharyngeal transit time

Author, year	Outcome measure	Bolus type	NGT	No NGT	P-value	Mean difference (95% CI)
Oral transit time						
Wang et al., 2006 [28]	Time (sec)	Thin barium	3.79±3.80	3.16±1.79	0.410	2.12 (0.48; 3.76)
Wang, Chen and Ni, 2019 [30]			1.69±0.21	1.67±0.21	1.548	0.02 (-0.09; 0.12)
Wang et al., 2006 [28]		Thick barium	5.33±4.00	4.82±3.70	0.580	3.60 (1.88; 5.32)
Wang, Chen and Ni, 2019 [30]			1.76±0.24	1.73±0.25	0.528	0.03 (-0.10; 0.16)
Nam et al., 2015 [34]	Functional Dysphagia Scale	35% diluted barium solution	1.95±2.85	1.50±2.63	0.080	0.45 (-0.77; 1.67)
Pharyngeal transit time						
Wang et al., 2006 [28]	Time (sec)	Thin barium	2.61±2.75	2.43±3.16	0.410	0.18 (-1.62; 1.98)
Wang, Chen and Ni, 2019 [30]			1.48±0.14	1.08±0.16	<0.001*	0.40 (0.32; 0.48)
Wang et al., 2006 [28]		Thick barium	3.09±2.20	2.79±2.52	0.260	0.30 (-1.14; 1.74)
Wang, Chen and Ni, 2019 [30]			1.51±0.17	1.09±0.16	<0.001*	0.42 (0.33; 0.51)
Nam et al., 2015 [34]	Functional Dysphagia Scale	35% diluted barium solution	2.30±2.00	2.10±2.02	0.160	0.20 (-0.69; 1.09)

Data are mean±SD total score unless indicated. NGT, nasogastric tube

**Table 6:** Summarised results for aspiration/penetration

Author, year	Bolus type	NGT	No NGT	*P*-value	Mean difference (95% CI)
*Videofluoroscopic Dysphagia Scale*					
Jung et al., 2021 [31]	Nectar	4.0 ± 5.1	2.6 ± 4.9	0.056	1.40 (-1.72; 4.52)
	Puree	2.9 ± 4.8	2.0 ± 4.4	0.186	0.90 (-1.97; 3.77)
*Penetration Aspiration Scale*					
Wang, Chen and Ni, 2019 [30]	Thin barium	1.8 ± 0.14	1.23 ± 0.62	< 0.001*	0.57 (0.34; 0.80)
	Thick barium	1.65 ± 0.15	1.77 ± 0.61	< 0.001*	-0.12 (-0.35; 0.11)
*Modified Penetration Aspiration Scale*					
Nam et al., 2015 [34]	35% diluted barium solution	2.40 ± 1.06	1.83 ± 0.98	< 0.001*	0.57 (0.12; 1.02)

Data are mean ± SD total score unless indicated. NGT, nasogastric tube

##### Pharyngeal Residue

Four studies reported on pharyngeal residues [27, 30, 31, 34]; three of these studies included statistical comparison of pharyngeal residue between conditions (Table 7) [30, 31, 34]. The remaining study compared outcomes descriptively and reported two of 25 participants had increased residue with an NGT compared to without, however this was “caused by a tube-related worsening of dysphagia by a mechanical interference of the NGT…” as it remained in the pyriform sinuses but was attached to the NGT [27; Results section, part 2, para. 1].

**Table 7:** Summarised results for pharyngeal residue

Author, year	Outcome measure	Bolus type	NGT	No NGT	*P*-value	Mean difference (95% CI)
*Vallecula residue*						
Nam et al., 2015 [34]	Functional Dysphagia Scale	35% diluted barium solution	3.80 ± 2.39	2.90 ± 2.22	0.002*	1.00 (-0.29; 2.29)
Wang, Chen and Ni, 2019 [30]	3-point scale	Thin barium	Nil data	Nil data	0.001*	N/A
		Thick barium	Nil data	Nil data	0.005*	N/A
*Pyriform sinus residue*						
Nam et al., 2015 [34]	Functional Dysphagia Scale	35% diluted barium solution	3.60 ± 3.11	2.60 ± 2.65	0.001*	0.90 (-0.13;1.93)
Wang, Chen and Ni, 2019 [30]	3-point scale	Thin barium	Nil data	Nil data	0.001*	N/A
		Thick barium	Nil data	Nil data	0.005*	N/A
Jung et al., 2021 [31]	Videofluoroscopic Dysphagia Scale	Nectar	3.4 ± 12.4	3.2 ± 4.5	0.329	0.20 (-2.61; 3.01)
		Puree	3.4 ± 4.5	3.2 ± 4.5	0.329	0.20 (-0.29; 2.29)

Data are mean ± SD total score unless indicated. NGT, nasogastric tube

##### Other Reported Outcomes

One study provided descriptive results (nil data provided) for ‘saliva pooling’ and ‘premature spillage’ and reported no difference between the conditions of NGT and no-NGT for participants with stroke [27]. As no measurable data were reported, this study was excluded from the quantitative synthesis.

## Discussion

### Summary of Findings

To our knowledge, this is the first systematic review to investigate the impact of NGTs on swallowing function in stroke survivors with dysphagia. Overall, this review found that research on this topic was limited in number and methodological quality. Of the eight studies included, five found differences on one or more aspect of swallowing when comparing NGT and no-NGT conditions. The risk of bias within the included studies was moderate to high, and therefore, the findings must be interpreted with caution. When patients post stroke have NGTs in situ the included articles indicate that there is:

- Greater overall dysphagia severity, particularly with fluids, as evidenced by significantly higher scores (*p* < 0.05) on the FDS, VDS and Wa Tian Water Drinking Test [30, 31, 34].
- Longer pharyngeal transit (seconds) (*p* < 0.001) [30].
- Increased severity of penetration/aspiration [30, 34] with higher PAS/mPAS scores on thin fluid trials (*p* < 0.001) and increased risk of aspiration due to reduced degree (*p* = 0.005) and distance (*p* = 0.009) of hyoid bone movement.
- Higher rates of pneumonia (*p* = 0.001) [29].
- Increased volume of pharyngeal residue, particularly with fluids [30, 34] on the FDS (*p* < 0.005) and another 3-point rating scale (*p* < 0.001).
- No evidence to suggest that the oral phase of swallowing was impacted by NGT insertion based on currently available evidence [28, 30, 34].

In summary, both bolus efficiency (pharyngeal residue and transit times) and airway protection (penetration/aspiration) may be impacted by NGTs in patients post stroke. Similar trends have been previously found in healthy older adults [13], but not in patients with dysphagia. Studies of dysphagic participants have included mixed dysphagia aetiologies (i.e. stroke in addition to head and neck surgery or progressive neurological diseases) and have generally only studied airway protection, with nil major NGT impacts reported [14, 15]. Further research is required to determine if the impacts of NGTs differ for neuromuscular dysphagia caused by a stroke compared to structural dysphagia caused by cancer or surgery.

#### Assessment Methodology

There were significant methodological differences between studies which likely contributed to the variation in findings as discussed below.

##### Swallow Assessment Method

The included studies employed various methods to assess changes in swallow function. Even when direct swallowing assessments such as VFSS or FEES were used, insufficient reporting of assessment procedures was common. This highlights the need for future studies to utilise a standardised VFSS protocol (such as the Modified barium swallow impairment profile; MBSImP) [38] to improve validity and generalisability of findings. Of the two studies that used indirect methods, one study [29] used a non-validated assessment to assess changes (the Wa Tian Water Test). This limited the methodological strength of the study as the diagnostic accuracy of this assessment is unknown. Conversely, Kwak et al. [33] utilised ultrasonography, which although indirect, recent research suggests that it that it has good sensitivity and specificity in detecting aspiration [39] and lingual dysfunction [40].

##### Food and Fluid Trials

There was substantial methodological heterogeneity in the bolus types and volumes used across studies. No single bolus type was consistently trialled, and where there was overlap (i.e. two studies [31, 37]) reported trials of ‘puree’), the viscosity was neither defined nor quantified, limiting comparability. This made direct comparisons of swallowing between studies difficult given the known differences in swallowing timing and kinematics between various food and fluid viscosities [17]. This underscores the need for future studies to reference the 2012 International Dysphagia Diet Standardisation Initiative (IDDSI) [36], which provides consistent terminology and testing methods to ensure adequate replicability and translation to clinical practice. Sip volumes also differed markedly. Four studies limited sip volume to 5mL or less although new evidence suggests that average volume for thin fluids may be approximately 12mL [17]. Hence, reported results of these studies may not be reflective of real-world swallowing behaviour.

##### Timing of Swallowing Assessment

There were insufficient data to complete further subgroup analysis of the time between NGT removal and repeat swallowing assessment. Whilst five studies reported on the time intervals between pre- and post-assessments, two did not [32, 33] indicating a potential source of measurement bias. Two studies left a five-hour gap between assessments and reported significant differences in overall swallowing function and penetration/aspiration between conditions [30, 34]. Interestingly, participants in both these studies also had a mean NGT insertion of greater than 65 days, significantly longer than the other included studies. Both studies suggested that long-term NGT insertion could impact pharyngeal coordination due to oedema, pain and desensitisation, which is consistent with previous research conducted on patients with dysphagia one year post stroke [2]. However, it should be noted that causality should not be inferred given the observational designs of the studies. The authors recommended a period of rest after NGT removal to reduce its impact on swallowing. However, it is unclear to what degree the prolonged NGT use impacted these results. The lack of standardisation regarding NGT duration and rest intervals in the studies in this review is a critical gap in the literature. These differences act as cofounding variable and limit comparability across studies. Future research should aim to control for NGT duration to better isolate its effects on swallowing physiology.

##### Participant Selection

A potential bias in participant selection was noted in three studies [28, 30, 31, 34]; these specified exclusion criteria around aspiration despite including aspiration as an outcome measure. Furthermore, two of these studies also included pharyngeal residue as an outcome of interest [30, 31], however, severe aspiration and pharyngeal residue have been shown to be positively correlated [41, 42]. Therefore, participant selection may have been biased towards those with mild to moderate dysphagia, given aspiration is a marker of severe dysphagia [43]. Generalisability of the findings may also have been impacted by the significant variation in aspiration criteria and inclusion/exclusion criteria more generally. Additionally, six out of the eight [28–33] included studies did not report how dysphagia was initially diagnosed, representing a substantial methodological limitation. This lack of transparency increases the risk of undetected selection bias. Furthermore, only one study provided sample size justification and power calculations limiting confidence in the statistical robustness of the findings.

#### Limitations of the Evidence and Systematic Review

These findings should be interpreted with caution as the quality of the evidence was affected by high risk of bias ratings. Furthermore, reporting of methodological and participant details such as time since stroke, type of NGT, and how dysphagia was diagnosed was identified as a major inconsistency between studies. Pooling of data across studies was determined to be inappropriate given the significant heterogeneity seen in swallowing assessment methodology and measurement of outcomes as discussed previously. As the studies included in this review used a range of designs, the absence of inferential statistics in some studies [27, 28, 33] limits the ability comparing findings. Nonetheless, this systematic review was able to identify trends which can inform future research and clinical practice.

There were three limitations to this review. First, the population of interest was limited to people with stroke, however, this also resulted in only a small number of studies. Potentially, there may be relevant studies investigating the effect of NGT on swallowing function in other populations which may be clinically informative. Generalisability was also impacted by low methodological quality and heterogeneity between studies meant that meta-analysis was not appropriate. Secondly, a robust assessment of risk of bias was limited as there are a number of available critical appraisal tools for pre-post study designs. The JBI tool was ultimately selected for consistency of appraisal method across studies, however, not all questions in this checklist were relevant to pre-post study designs. Future reviews may consider using alternative appraisal tools for assessing the risk of bias in non-randomised studies of interventions such as the ROBINS-I [44] (recommended by Cochrane Handbook for Systematic Reviews of Interventions [45]) or the NIH Quality Assessment Tool for Before/After Studies [46]. Additionally, the use of a binary scoring system to summarise responses may have oversimplified complex judgments and potentially overlooked important nuances in bias assessment. Lastly, this review applied a language restriction (English) and did not include a formal grey literature search, which may have introduced publication bias by excluding unpublished studies or those in non-English languages. Future reviews could consider a broader search strategy, including grey literature sources and formal publication bias assessments, to improve comprehensiveness.

#### Clinical Recommendations

This study represents a first step in summarising the impacts of NGTs on stroke patients with dysphagia and provides some preliminary implications for clinical practice. NGT removal is contingent on meeting nutritional needs orally, however our findings raise the possibility that NGT placement may impact swallowing efficiency and safety, which in turn may prolong its own necessity. This hypothesis warrants further exploration in future longitudinal studies. In the interim, although nutrition optimisation falls outside the scope of Speech Pathology practice, clinicians should ensure that nutritional needs are carefully balanced with swallowing recovery to ensure optimal patient outcomes. It is therefore crucial for Speech Pathologists to monitor the effects of the NGT and work collaboratively with the multidisciplinary team when managing dysphagia.

Our findings raise the possibility that NGT placement may impact swallowing efficiency and safety, which in turn may prolong its own necessity. This hypothesis warrants exploration in future longitudinal studies.

Temporary removal of the NGT during instrumental swallowing assessment could be trialled, provided the patient has a functional swallow, is medically stable and nutritional and/or pharmacological needs will not be compromised. NGT removal would allow clinicians to differentiate between effects of the stroke and structural interference from the NGT on swallowing function. This would ensure that clinicians are not over-prescribing swallowing rehabilitation exercises for deficits that are likely to improve spontaneously once the NGT is removed. For example, one study found residue in the pyriform sinuses with the NGT in situ that was not present at baseline and attributed this to mechanical interference from the NGT; material became stuck to the NGT and remained in the pyriform sinus post swallow [27]. Another found that removing the NGT resulting in hyoid function almost immediately being restored to the same levels as their control group (stroke patients without NGT) [33]. However, temporary NGT removal and reinsertion also poses its own risks (e.g. missed medications, discomfort, incorrect placement and trauma to the nose, pharynx and oesophagus [47, 48]). Therefore, further research is first needed to establish evidence-based criteria for temporary NGT removal to investigate the safety, feasibility and treatment impact of such protocols.

Furthermore, the utility of long-term NGT use may need to be considered with alternative options of enteral feeding. Two studies included in this review had an average NGT insertion time of greater than two months [30, 34]. Both studies found significant differences in pharyngeal function once the NGT was removed, suggesting that the NGT was contributing to the patients’ swallowing impairments. Further research is required to investigate whether alternative means of enteral nutrition may be more appropriate for patients whose swallowing recovery is expected to be slow and therefore may require longer-term nutritional support.

#### Future Directions

The absence of high-quality evidence is a critical finding of the review and highlights a need for further methodologically vigorous research to improve the quality and generalisability of results. Future studies should consider the use of prospective and representative recruitment of participants, as well as valid, reliable and replicable assessment methods. Specifically, the following should be considered:

1. Studies should report in detail the method and criteria used to diagnose dysphagia in stroke survivors included in the study. Ideally, an assessment should be conducted by a Speech Pathologist (or equivalent) using pre-determined and normative-based diagnostic criteria. In addition, studies should report interrater reliability if multiple assessors are involved;
2. Thorough description of the assessment methods is needed to allow for replicability of the study. This should include an assessment protocol with detail on volumes and viscosities trialled, the order of bolus presentation and the number of swallowing trials per bolus type. Bolus viscosities should be described using the terminology outlined by the International Dysphagia Diet Standardisation Initiative (IDDSI) Framework to allow for universal comparison; 
3. Where possible, direct assessment of the swallow should be conducted to determine differences between groups and/or conditions. Evidence suggests that VFSS and FEES are currently the gold standard in accurately assessing the pharyngeal swallow, as it allows for direct visualisation of any aspiration or residue [43];
4. Valid and reliable methods should be used to compare swallowing function between conditions, either using validated rating scales or quantitative measures. Sufficient detail of the rating and/or measuring criteria should be reported;
5. Studies should provide more comprehensive participant-level data to allow for more nuanced understanding of NGT effects across different clinical profiles. This includes reporting on variables such as time since stroke onset, duration of NGT placement, and any concurrent dysphagia rehabilitation received;
6. Future studies should explicitly report sample size justification and power calculations to ensure adequate statistical rigour;
7. Longitudinal studies designs may also be informative in determining whether swallowing impairments associated with NGT presence are reversible once the tube is removed. This would clarify whether observed physiological changes are temporary or indicative of longer-term dysfunction;
8. Future research using a qualitative or mixed-methods approach may complement our findings by addressing patient perspectives and contextual factors influencing care; and
9. While this study focused primarily on the physiological metrics, it is important that future studies consider swallowing physiology in the context clinically meaningful patient outcomes, such as nutrition/dehydration, aspiration pneumonia, and lived experiences of patients such as their quality of life.

## Conclusion

A key finding of this review is the lack of high-quality evidence investigating the impact of NGTs on swallowing function for stroke survivors. The findings suggest NGTs may negatively impact swallowing function on one or more swallowing parameters, with the pharyngeal stage of the swallow appearing most impacted. However, the presence of moderate to high risk of bias and inconsistencies in methodology between included studies limit the reliability and generalisability of the findings. In the future, more methodologically robust research is warranted to guide dysphagia management in stroke survivors requiring an NGT.

## Supplementary Information

Below is the link to the electronic supplementary material.

Supplementary Material 1

Supplementary Material 2

Supplementary Material 3

## Declarations

### Data Accessibility

Data sharing is not applicable to this article as no datasets were generated during the current study.
