# Supplementary material for: The Impact of Nasogastric Tubes (NGTs) on Swallow Function in Adults with Post-stroke Dysphagia: A Systematic Review
Source: Dysphagia. 2026 Mar 24;41(4):745–58. doi: 10.1007/s00455-026-10926-7 (PMC13457347; doi:10.1007/s00455-026-10926-7)
Supplement: Supplementary file 1 [file 455_2026_10926_MOESM1_ESM.docx]

**Appendix 1** Search strategy for each database

**EBSCO CINAHL**

| **#** | **Query** | **Results from 11 Jan 2023** |
| --- | --- | --- |
| S1 | (MH "Nasoenteral Tubes") OR (MH "Intubation, Gastrointestinal") OR (MH "Feeding Tubes") OR (MH "Jejunostomy Tubes") OR (MH "Gastrostomy Tubes") OR (MH "Gastrojejunostomy Tubes") | 4,922 |
| S2 | (nasogastric or “naso gastric”) N2 (tube? or feeding or fed or intubat*) | 2,336 |
| S3 | (nasoenteral or “naso enteral”) N2 (tube? or feeding or fed or intubat*) | 1,081 |
| S4 | NGT N2 (tube? or feeding or fed or intubat*) | 181 |
| S5 | NG N2 (tube? or feeding or fed or intubat*) | 268 |
| S6 | (Nasojejunal or “naso jejunal”) N2 (tube? or feeding or fed or intubat*) | 173 |
| S7 | Intubat* N2 (gastrointestinal* or gastro intestinal* or nasogastric* or naso gastric* or nasojejunal* or naso jejunal*) | 2,341 |
| S8 | (Fed or feeding) N2 (enteral* or tube? Or gastric or force) | 7,537 |
| S9 | enteral N2 (fed or intubat*) | 86 |
| S10 | S1 OR S2 OR S3 OR S4 OR S5 OR S6 OR S7 OR S8 OR S9 | 11,143 |
| S11 | (MH "Deglutition Disorders") OR (MH "Swallowing Therapy") | 10,633 |
| S12 | aphagopraxi* or dysphagi* | 10,864 |
| S13 | deglutition N2 (difficult* or disorder* or d#sfunction or problem* or function) | 9,999 |
| S14 | swallow* N2 (difficult* or disorder* or d#sfunction or problem* or function) | 3,884 |
| S15 | S11 OR S12 OR S13 OR S14 | 16,777 |
| S16 | (MH "Cerebral Infarction") OR (MH "Hemorrhagic Stroke") OR (MH "Ischemic Stroke") OR (MH "Embolic Stroke") OR (MH "Stroke, Lacunar") OR (MH "Subarachnoid Hemorrhage") OR (MH "Stroke") OR (MH "Hematoma, Subdural, Chronic") OR (MH "Hematoma, Subdural, Acute") OR (MH "Hematoma, Subdural") OR (MH "Hematoma, Epidural") OR (MH "Basal Ganglia Hemorrhage") OR (MH "Cerebral Hemorrhage") OR (MH "Intracranial Hemorrhage") OR (MH "Intracranial Embolism") OR (MH "Intracranial Thrombosis") OR (MH "Sinus Thrombosis, Intracranial") OR (MH "Cerebral Ischemia") | 100,610 |
| S17 | stroke? Or apoplexi* or CVA? | 142,113 |
| S18 | Cerebr* or brain or intracerebr* N2 (stroke? Or accident? or apoplex* or isch?emi* or insult* or fail* or injur* or insuffici* or h*mmorhag* or occlu* or infact*) | 315,451 |
| S19 | Stroke? N2 (cerebr* or intracerebr* or acute or vascular or apoplexi*) | 19,825 |
| S20 | S16 OR S17 OR S18 OR S19 | 419,154 |
| S21 | S10 AND S15 AND S20 | 251 |

**Ovid Embase Classic+Embase <1947 to 2023 January 09>** 
 

| **#** | **Query** | **Results from 11 Jan 2023** |
| --- | --- | --- |
| 1 | nasogastric tube/ | 15,099 |
| 2 | ((nasogastric or naso gastric) adj2 (tube? or feeding or fed or intubat*)).mp. | 20,222 |
| 3 | ((nasoenteral or naso enteral) adj2 (tube? or feeding or fed or intubat*)).mp. | 250 |
| 4 | (NGT adj2 (tube? or feeding or fed or intubat*)).mp. | 829 |
| 5 | (NG adj2 (tube? or feeding or fed or intubat*)).mp. | 1,556 |
| 6 | ((Nasojejunal or naso jejunal) adj2 (tube? or feeding or fed or intubat*)).mp. | 1,057 |
| 7 | (Intubat* adj2 (gastrointestinal* or gastro intestinal* or nasogastric* or naso gastric* or nasojejunal* or naso jejunal*)).mp. | 907 |
| 8 | ((Fed or feeding) adj2 (enteral* or tube? or gastric or force)).mp. | 26,591 |
| 9 | (enteral adj2 (fed or intubat*)).mp. | 207 |
| 10 | 1 or 2 or 3 or 4 or 5 or 6 or 7 or 8 or 9 | 44,606 |
| 11 | dysphagia/ or swallowing/ | 108,818 |
| 12 | (aphagopraxi* or dysphagi*).mp. | 100,550 |
| 13 | (deglutition adj2 (difficult* or disorder* or d#sfunction or problem* or function)).mp. | 3,837 |
| 14 | (swallow* adj2 (difficult* or disorder* or d#sfunction or problem* or function)).mp. | 15,832 |
| 15 | 11 or 12 or 13 or 14 | 122,425 |
| 16 | cerebrovascular accident/ or brainstem stroke/ or cardioembolic stroke/ or ischemic stroke/ or lacunar stroke/ | 286,446 |
| 17 | ischemic stroke/ or acute ischemic stroke/ or anterior circulation stroke/ or chronic ischemic stroke/ or cryptogenic ischemic stroke/ or posterior circulation stroke/ or subacute ischemic stroke/ or wake up stroke/ | 20,935 |
| 18 | basal ganglion hemorrhage/ or putaminal hemorrhage/ | 984 |
| 19 | anterior circulation infarction/ or partial anterior circulation infarct/ or total anterior circulation infarct/ | 105 |
| 20 | brain infarction/ or brain stem infarction/ or cadasil/ or cerebellum infarction/ or lacunar infarction/ or migrainous infarction/ or posterior circulation infarction/ | 68,283 |
| 21 | posterior circulation ischemia/ or posterior circulation infarction/ or posterior circulation stroke/ | 398 |
| 22 | brain ischemia/ or anterior circulation ischemia/ or brain vasospasm/ or posterior circulation ischemia/ | 163,973 |
| 23 | occlusive cerebrovascular disease/ or basilar artery obstruction/ or middle cerebral artery occlusion/ | 35,383 |
| 24 | (stroke? or apoplexi* or CVA?).mp. | 561,971 |
| 25 | ((Cerebr* or brain or intracerebr*) adj2 (stroke? or accident? or apoplex* or isch?emi* or insult* or fail* or injur* or insuffici* or h*mmorhag* or occlu* or infact*)).mp. | 620,475 |
| 26 | (Stroke? adj2 (cerebr* or intracerebr* or acute or vascular or apoplexi*)).mp. | 83,225 |
| 27 | 24 or 25 or 26 | 881,105 |
| 28 | 10 and 15 and 27 | 878 |

 
**Ovid Emcare**

| **#** | **Query** | **Results from 11 Jan 2023** |
| --- | --- | --- |
| 1 | nasogastric tube/ | 3,015 |
| 2 | ((nasogastric or naso gastric) adj2 (tube? or feeding or fed or intubat*)).mp. | 4,412 |
| 3 | ((nasoenteral or naso enteral) adj2 (tube? or feeding or fed or intubat*)).mp. | 67 |
| 4 | (NGT adj2 (tube? or feeding or fed or intubat*)).mp. | 203 |
| 5 | (NG adj2 (tube? or feeding or fed or intubat*)).mp. | 247 |
| 6 | ((Nasojejunal or naso jejunal) adj2 (tube? or feeding or fed or intubat*)).mp. | 234 |
| 7 | (Intubat* adj2 (gastrointestinal* or gastro intestinal* or nasogastric* or naso gastric* or nasojejunal* or naso jejunal*)).mp. | 193 |
| 8 | ((Fed or feeding) adj2 (enteral* or tube? or gastric or force)).mp. | 7,749 |
| 9 | (enteral adj2 (fed or intubat*)).mp. | 73 |
| 10 | 1 or 2 or 3 or 4 or 5 or 6 or 7 or 8 or 9 | 11,347 |
| 11 | dysphagia/ or swallowing/ | 20,457 |
| 12 | (aphagopraxi* or dysphagi*).mp. | 21,251 |
| 13 | (deglutition adj2 (difficult* or disorder* or d#sfunction or problem* or function)).mp. | 1,559 |
| 14 | (swallow* adj2 (difficult* or disorder* or d#sfunction or problem* or function)).mp. | 4,443 |
| 15 | 11 or 12 or 13 or 14 | 26,273 |
| 16 | cerebrovascular accident/ or brainstem stroke/ or cardioembolic stroke/ or ischemic stroke/ or lacunar stroke/ | 45,792 |
| 17 | ischemic stroke/ or acute ischemic stroke/ or anterior circulation stroke/ or chronic ischemic stroke/ or cryptogenic ischemic stroke/ or posterior circulation stroke/ or subacute ischemic stroke/ or wake up stroke/ | 678 |
| 18 | basal ganglion hemorrhage/ or putaminal hemorrhage/ | 145 |
| 19 | anterior circulation infarction/ or partial anterior circulation infarct/ or total anterior circulation infarct/ | 7 |
| 20 | brain infarction/ or brain stem infarction/ or cadasil/ or cerebellum infarction/ or lacunar infarction/ or migrainous infarction/ or posterior circulation infarction/ | 11,534 |
| 21 | posterior circulation ischemia/ or posterior circulation infarction/ or posterior circulation stroke/ | 30 |
| 22 | brain ischemia/ or anterior circulation ischemia/ or brain vasospasm/ or posterior circulation ischemia/ | 23,443 |
| 23 | occlusive cerebrovascular disease/ or basilar artery obstruction/ or middle cerebral artery occlusion/ | 5,273 |
| 24 | (stroke? or apoplexi* or CVA?).mp. | 140,030 |
| 25 | ((Cerebr* or brain or intracerebr*) adj2 (stroke? or accident? or apoplex* or isch?emi* or insult* or fail* or injur* or insuffici* or h*mmorhag* or occlu* or infact*)).mp. | 116,369 |
| 26 | (stroke? adj2 (cerebr* or intracerebr* or acute or vascular or apoplexi*)).mp. | 20,143 |
| 27 | 16 or 17 or 18 or 19 or 20 or 21 or 22 or 23 or 24 or 25 or 26 | 210,132 |
| 28 | 10 and 15 and 27 | 262 |

**Ovid MEDLINE(R) <1946 to January 09, 2023>** 

| **#** | **Query** | **Results from 11 Jan 2023** |
| --- | --- | --- |
| 1 | Intubation, Gastrointestinal/ or Enteral Nutrition/ | 29,296 |
| 2 | ((nasogastric or naso gastric) adj2 (tube? or feeding or fed or intubat*)).mp. | 6,222 |
| 3 | ((nasoenteral or naso enteral) adj2 (tube? or feeding or fed or intubat*)).mp. | 151 |
| 4 | (NGT adj2 (tube? or feeding or fed or intubat*)).mp. | 360 |
| 5 | (NG adj2 (tube? or feeding or fed or intubat*)).mp. | 596 |
| 6 | ((Nasojejunal or naso jejunal) adj2 (tube? or feeding or fed or intubat*)).mp. | 451 |
| 7 | (Intubat* adj2 (gastrointestinal* or gastro intestinal* or nasogastric* or naso gastric* or nasojejunal* or naso jejunal*)).mp. | 10,455 |
| 8 | ((Fed or feeding) adj2 (enteral* or tube? or gastric or force)).mp. | 13,942 |
| 9 | (enteral adj2 (fed or intubat*)).mp. | 114 |
| 10 | 1 or 2 or 3 or 4 or 5 or 6 or 7 or 8 or 9 | 38,585 |
| 11 | Deglutition Disorders/ | 23,268 |
| 12 | (aphagopraxi* or dysphagi*).mp. | 28,691 |
| 13 | (deglutition adj2 (difficult* or disorder* or d#sfunction or problem* or function)).mp. | 23,583 |
| 14 | (swallow* adj2 (difficult* or disorder* or d#sfunction or problem* or function)).mp. | 7,616 |
| 15 | 11 or 12 or 13 or 14 | 41,414 |
| 16 | basal ganglia cerebrovascular disease/ or basal ganglia hemorrhage/ or putaminal hemorrhage/ or brain ischemia/ or brain infarction/ or brain stem infarctions/ or lateral medullary syndrome/ or infarction, anterior cerebral artery/ or infarction, middle cerebral artery/ or infarction, posterior cerebral artery/ or stroke/ or hemorrhagic stroke/ or ischemic stroke/ or embolic stroke/ or thrombotic stroke/ or stroke, lacunar/ | 176,870 |
| 17 | (stroke? or apoplexi* or CVA?).mp. | 326,667 |
| 18 | ((Cerebr* or brain or intracerebr*) adj2 (stroke? or accident? or apoplex* or isch?emi* or insult* or fail* or injur* or insuffici* or h*mmorhag* or occlu* or infact*)).mp. | 203,629 |
| 19 | (Stroke? adj2 (cerebr* or intracerebr* or acute or vascular or apoplexi*)).mp. | 39,083 |
| 20 | 16 or 17 or 18 or 19 | 471,397 |
| 21 | 10 and 15 and 20 | 389 |
