# Supplementary material for: The Impact of Nasogastric Tubes (NGTs) on Swallow Function in Adults with Post-stroke Dysphagia: A Systematic Review
Source: Dysphagia. 2026 Mar 24;41(4):745–58. doi: 10.1007/s00455-026-10926-7 (PMC13457347; doi:10.1007/s00455-026-10926-7)
Supplement: Supplementary file 2 [file 455_2026_10926_MOESM2_ESM.docx]

**Electronic Supplementary Material 1** Reported outcomes and outcome measures

**Penetration/ Aspiration**

DIRECT MEASURES

- Penetration Aspiration Scale (PAS) [35]: an 8-point scale reflecting 1) the depth of airway entry (material above, contacting, or below the level of vocal folds; (2) how effectively the material was cleared from the airway (ejected/not ejected); and (3) the patient’s sensory response to material present in the airway (attempts to clear made/not made). A higher score indicates a greater level of impairment [30-32]
- Modified PAS (mPAS): a modified 5-point version of PAS scale [27, 34]
- Binary measure (present/absent) [28]

INDIRECT MEASURES

- Incidence of stroke-associated pneumonia: an adverse clinical outcome of aspiration [39] caused by bacteria and/or ingested material entering the lungs and assessed via chest x-rays in combination with ≥2 pre-defined clinical markers of infection [29]
- Hyoid bone movement: an abnormal decreased or delay in hyoid bone movement is considered to be “a major risk factor for aspiration” [33]

**Swallow transit times**

- Seconds [28, 30]
- Functional Dysphagia Scale (FDS) [36]: validated rating scale comprised of numerical ratings for individual swallowing parameters to derive a total score out of 100 (higher score indicates greater level of impairment) [34]
  - Oral transit time: allocated score 0 <1.5 sec, score 6 >1.5 sec
  - Pharyngeal transit time: allocated score 0 <1.0 sec, score 4 > 1.0 sec

**Overall swallow function**

- FDS [30, 34]
- Videofluoroscopic Dysphagia Scale (VDS) [37]: as per FDS [31]
- Wa Tian water drinking test: a 5-point scale that describes the number of swallows and whether coughing observed after drinking 30mL of water (level 5 indicates a greater level of impairment) [29]

**Pharyngeal residue**

- VDS/FDS rating scale; proportion (%) of vallecular and pyriform structures remained filled with barium post swallow [31, 34].
- 3-point scale: graded on 0–3 scale (0 = no residue, 1 = residue <25% of structure height , 2 = residue 25 to 50%, 3 = residue >50%) [30]
- Not specified [27]
