# Supplementary material for: The Impact of Nasogastric Tubes (NGTs) on Swallow Function in Adults with Post-stroke Dysphagia: A Systematic Review
Source: Dysphagia. 2026 Mar 24;41(4):745–58. doi: 10.1007/s00455-026-10926-7 (PMC13457347; doi:10.1007/s00455-026-10926-7)
Supplement: Supplementary file 3 [file 455_2026_10926_MOESM3_ESM.docx]

**Electronic Supplementary Material 2** Summary of reported assessment methodology

| **Study** | **Swallow assessment method** | **Reported outcomes** | | | | | | **Time between measures (hours)** | **Reported food and/or fluids trials** |
| --- | --- | --- | --- | --- | --- | --- | --- | --- | --- |
|  |  | Penetration/aspiration | Oral transit time | Pharyngeal transit time | Pharyngeal residue (vallecula and/or pyriform sinuses) | Overall swallow function | Other |  |  |
|  | | | | | | | | | |
| Wang et al., 2006 [28] | VFSS | Present/ absent | sec | sec | x | x | Swallow trigger time (sec) | 0.5 | 5mL thin fluids |
|  |  |  |  |  |  |  | Aspiration (present/absent) |  | 5mL thick fluids |
|  |  |  |  |  |  |  |  |  | Paste^1^ |
| Nam et al., 2015 [34] | VFSS | mPAS | FDS | FDS | FDS | FDS | x | 5 | 5mL of 35% diluted barium solution |
| Kim et al., 2018 [32] | VFSS | PAS | x | x |  | x | x | Not stated | 1mL thin fluids |
|  |  |  |  |  |  |  |  |  | Additional consistencies^b^ |
| Wang, Chen and Ni, 2019 [30] | VFSS | PAS | sec | sec | 3-point severity scale | FDS | x | 5 | 5mL thin barium sulphate (IDDSI Level 0 [39]) |
|  |  |  |  |  |  |  |  |  | 5mL thick barium sulphate (IDDSI Level 3 [39]) |
| Jung et al., 2021 [31] | VFSS | PAS | x | x | VDS | x | x | <1^a^ | Nectar |
|  |  |  |  |  |  |  |  |  | Puree |
|  | | | | | | | | | |
| Dziewas et al., 2008 [27] | FEES | mPAS | x | x | Not reported | x | Saliva pooling, premature spillage (not reported) | <1 | Puree |
|  |  |  |  |  |  |  |  |  | Liquids (n=21) |
|  |  |  |  |  |  |  |  |  | Soft solid food (n=18) |
|  | | | | | | | | | |
| Kwak et al., 2018 [33] | Ultrasonography | x | x | x |  | x | Hyoid bone movement (distance [cm], area [pixels], time [sec]) | Not stated | 1mL water |
|  | | | | | | | | | |
| Juan et al., 2020 [29] | Non-instrumental swallowing assessment | x | x | x |  | x | Incidence of stroke-associated pneumonia (pre-defined criteria) | N/A | 30mL warm water |
|  |  |  |  |  |  |  | Swallowing function (Wa Tian drinking water test) |  |  |
| VFSS, videofluroscopic swallow study; FEES, fibreendoscopic evaluations of swallowing. ^a^ Authors reported pre and post swallowing assessments were conducted ‘consecutively’. ^b^Only trialled in no-NGT condition (therefore no comparison available). | | | | | | | | | |
